# Effect of elbow carrying angle on lateral epicondylitis development

**DOI:** 10.1097/MD.0000000000035789

**Published:** 2023-10-27

**Authors:** Hakan Yolaçan, Serkan Güler

**Affiliations:** a Aksaray Training and Research Hospital, Orthopaedics and Traumatology, Aksaray, Turkey.

**Keywords:** cubitus varus, elbow carrying angle, lateral epicondylitis

## Abstract

Lateral epicondylitis is one of the most common elbow joint injuries and elbow anatomy is a risk factor. Our study aims to evaluate whether the elbow carrying angle affects the development of lateral epicondylitis by considering sex differences. Elbow radiographs of 211 people (aged 18–65 years) diagnosed with lateral epicondylitis and having anteroposterior radiographs of the elbow taken in the appropriate position in the imaging archive of our hospital between January 1, 2021 and January 1, 2022 were retrospectively analyzed. The control group comprised elbow radiographs of 113 people in the same age range. The study evaluated the age, sex, and side and elbow carrying angles of the participants in the patient and control groups. The average elbow carrying angle was calculated as 14.6 (7.8–22.1). No significant relationship was found between the lateral epicondylitis and control groups based on sex (*P* = .383), side (*P* = .634) and age (*P* = .189). The mean elbow carrying angle was 13.8 ± 3.7 in the group with lateral epicondylitis and 15.9 ± 3.6 in the control group and was significantly lower in the group with lateral epicondylitis (*P* < .05). A decrease in the elbow carrying angle namely cubitus varus, may lead to the development of lateral epicondylitis.

## 1. Introduction

Lateral epicondylitis is common in the working population and is a typical cause of disease-related workforce loss worldwide.^[1]^ It is one of the most common pathologies of the elbow. It is characterized by pain on the lateral side of the elbow joint, which occurs as a result of continuous and repetitive use of the wrist extensor muscles.^[2]^ Torque injury caused by overload and varus loading in the insertion of the extensor carpi radialis brevis (ECRB) muscle is the leading cause of the pathology. Although less common, extensor carpi radialis longus, extensor digitorum communis and pronator teres are also affected.^[3]^ It is seen in 1% to 3% of the population and is usually encountered between the ages of 30 and 60 years. Generally, it is more common in women, and the dominant extremity is more affected.^[4,5]^

Elbow anatomy is a risk factor for injury. The elbow carrying angle is the angle between the long axis of the humerus and that of the forearm when the forearm is in extension and supination. This angle is usually 14 to 16 degrees, differs according to sex and is generally greater in women than in men.^[6]^

The aim of our study is to evaluate whether the elbow carrying angle has an effect on the development of lateral epicondylitis by considering the sex difference.

The hypothesis of our study is that the decrease in elbow carrying angle namely cubitus varus is one of the factors causing the development of lateral epicondylitis.

## 2. Methods

**Figure 1.**
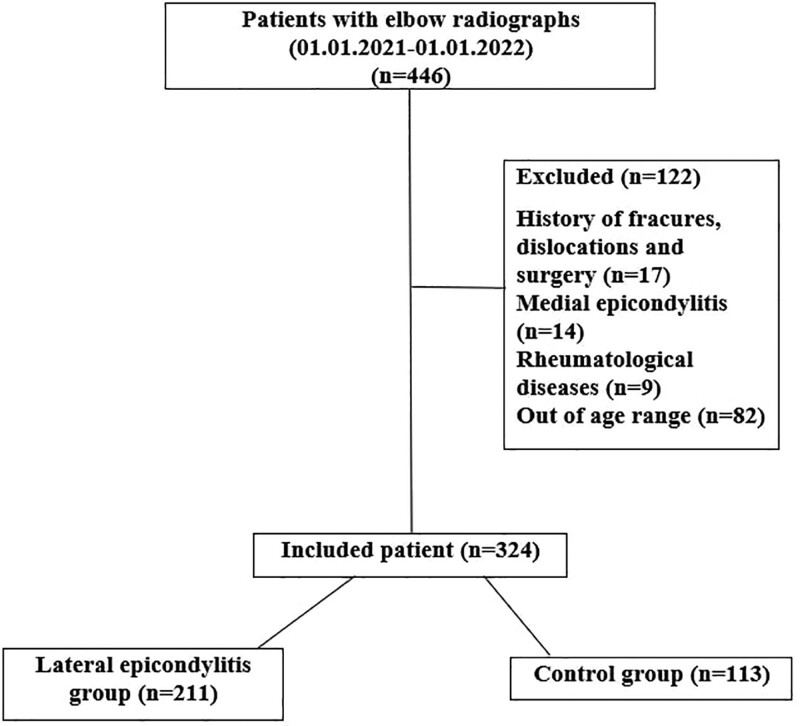
Flowchart of the study.

This study was approved by local ethics committee. In our study, patients who applied to the orthopedics and traumatology outpatient clinic of our hospital between January 1, 2021 and January 1, 2022 with complaints of pain in the elbow were evaluated. Patients with positive Cozen test used in the diagnosis of lateral epicondylitis and patients with elbow radiographs in the hospital imaging archive were retrospectively analyzed. Two hundred eleven patients aged 18 to 65 years who were diagnosed with lateral epicondylitis after examination and imaging were included in the study. The control group consisted of 113 people in the same age group who were not diagnosed with lateral epicondylitis and who had radiographs of the elbow for another reason. Patients with a history of fractures, dislocations and surgery in the evaluated upper extremity, patients with medial epicondylitis and those with rheumatological diseases were excluded from the study (Fig. 1).

**Figure 2.**
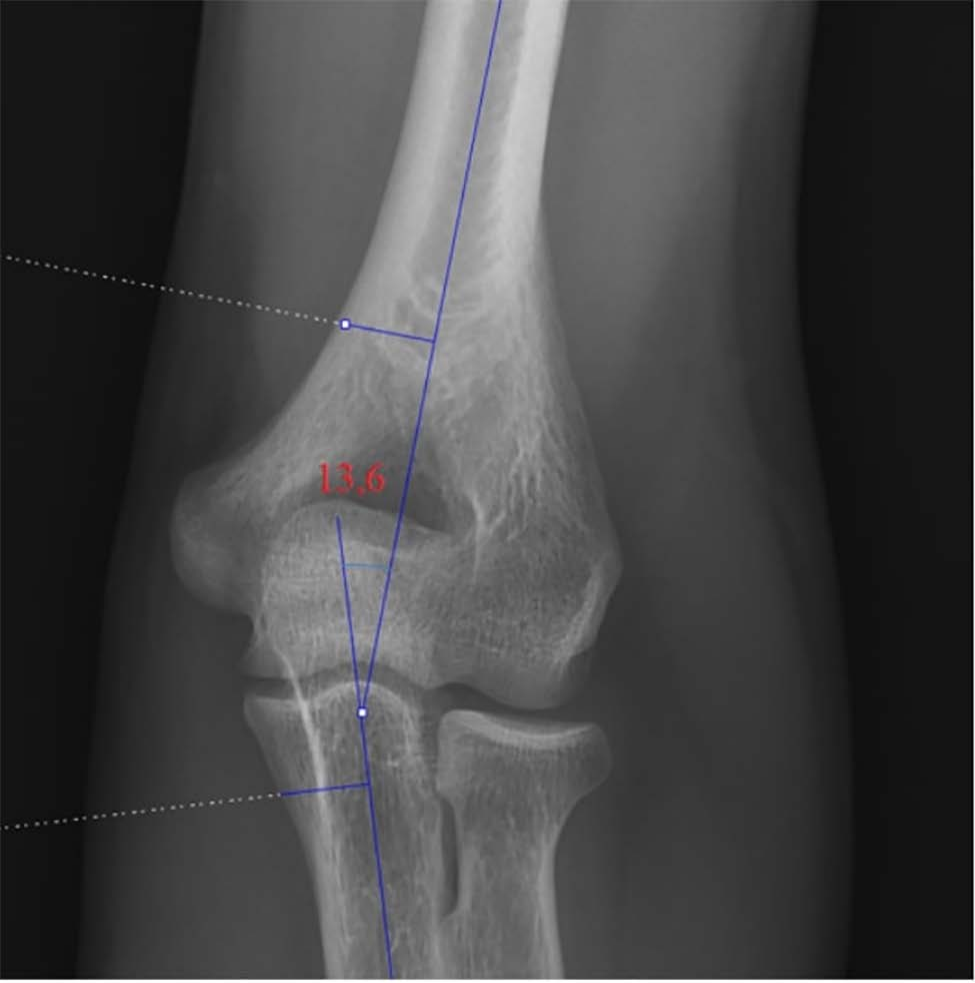
Demonstration of the measurement of elbow carrying angle on an elbow AP radiograph. AP = anteroposterior.

An abnormal increase in elbow carrying angle is called cubitus valgus, and an abnormal decrease is called cubitus varus. Measurements were made using the PACS system to view radiographs. Elbow carrying angle measurement was performed on radiography of the angle between the long axes of the humerus and ulna as described by Alsubael and Hegazy.^[7]^ Our study performed elbow carrying angle measurements on elbow anteroposterior radiographs (Fig. 2). The values on the radiograph were measured by 2 experienced orthopedic surgeons. Two weeks later, the same surgeons repeated the measurements. Inter- and intraobserver reliability were measured for radiographic measurements using intraclass correlation coefficients (ICCs) calculated from 2 sets of repeat measurements on a sample of 324 radiographs. The following scores were used: ICC > 0.80 indicates excellent; 0.70 to 0.80 indicates very good; 0.60 to 0.70 indicates good; 0.40 to 0.60 indicates fair; and 0.40 indicates poor.

The study evaluated the age, sex, side, and elbow carrying angles of the participants in the lateral epicondylitis and control groups.

### 2.1. Statistical analysis

The statistical data obtained were evaluated using the programme İBM SPSS Statistics (IBM SPSS Statistics for Windows, Version 26.0, Armonk, NY). The distribution homogeneity between the groups was evaluated with the Kolmogorov–Smirnov test. The mean values between groups were analyzed with the independent-samples *t* test at a significance level of 0.05.

## 3. Results

Of the participants in the study, 211 constituted the lateral epicondylitis group, and 113 constituted the control group; 167 were men and 157 were women. The left elbow was evaluated in 129 cases and the right elbow in 195 cases. The mean elbow carrying angle was calculated as 14.6 ± 3.8 (7.8–22.1). No significant difference was found between the groups based on sex (*P* = .383), side (*P* = .634) and age (*P* = .189) (Table 1).

**Table 1:** Comparison of the lateral epicondylitis and control groups based on sex, side, age and elbow carrying angle.

	Lateral epicondylitis	Control	*P* value
Number of elbows	211	113	
Sex, male: female	105:106	62:51	.383
Side, right:left	129:82	66:47	.634
Age (yr), average	42.3 ± 9.0	43.8 ± 10.5	.189
Elbow carrying angle, mean	13.8 ± 3.7	15.9 ± 3.6	<.001

The mean elbow carrying angle was 15.7 ± 3.7 in women and 13.5 ± 3.6 in men. A significant correlation was found between sex and elbow carrying angle according to the *P* < .05 significance level although the correlation was significantly lower in males (*P* < .001) (Table 1). Because the sex distribution between the groups was homogeneous, the lower elbow carrying angle in men did not affect our results.

The mean elbow carrying angle was 13.8 ± 3.7 in the group with lateral epicondylitis and 15.9 ± 3.6 in the control group, and the elbow carrying angle was significantly lower in the group with lateral epicondylitis (*P* < .05) (Table 1).

When intra and interobserver correlations were evaluated, we discovered that angle measurements exhibited high interobserver agreement (ICC, 0.91; 95% confidence interval, 0.89–0.96) and high intraobserver agreement (ICC, 0.93; 95% confidence interval, 0.91–0.98) (ICC, 0.91; 95% confidence interval, 0.88–0.94).

## 4. Discussion

The most important result of our study is that although the etiology of lateral epicondylitis is multifactorial, the reduced elbow carrying angle evaluated radiologically is associated with lateral epicondylitis.

Our study found no significant difference between the group diagnosed with lateral epicondylitis and the control group according to sex, side and age variables.

The relationship between sex and elbow carrying angle is controversial. In some studies, elbow carrying angle was higher in women than in men.^[8–11]^ In some studies, no significant relationship was observed between sex and elbow carrying angle.^[12,13]^ In our study, the elbow carrying angle measured in women was significantly higher than that in men.

The distance of the ECRB muscle between the origo and musculotendinous junction is the center of the pathology in lateral epicondylitis. Increasing this distance will result in higher stress and accordingly the decrease in elbow carrying angle and shift to varus will cause additional stress on the ECRB.^[14]^ As a result, the decrease in elbow caryying angle causes an abrasive effect on the ECRB tendon.

In the study of Umur et al^[15]^, in which 62 patients were examined the relationship between lateral epicondylitis and elbow carrying angle was examined. As a result of the study, it was stated that the increase in elbow carrying angle was effective on lateral epicondylitis. This result contradicts with our study. According to the results of our study, the elbow carrying angle was significantly lower in the group diagnosed with lateral epicondylitis than in the control group. This situation inferred that the decrease in the elbow carrying angle namely the cubitus varus, may lead to the development of lateral epicondylitis. In addition, the results of our study are consistent with the above-mentioned mechanic and anatomical theory.

### 4.1. Study limitations

The most important limitations of our study are that it was retrospective and body mass index was not evaluated. In addition, the elbow carrying angle is affected by the elbow position. The advantage of our study is that the number of patients is higher than the previous study on this subject.

## 5. Conclusion

Our study investigated the relationship between the elbow carrying angle and lateral epicondylitis and demonstrated that a decrease in the elbow carrying angle may cause lateral epicondylitis.

## Author contributions

**Conceptualization:** Hakan Yolaçan, Serkan Güler.

**Data curation:** Hakan Yolaçan, Serkan Güler.

**Formal analysis:** Hakan Yolaçan.

**Investigation:** Hakan Yolaçan, Serkan Güler.

**Methodology:** Hakan Yolaçan, Serkan Güler.

**Project administration:** Hakan Yolaçan.

**Resources:** Hakan Yolaçan.

**Software:** Hakan Yolaçan, Serkan Güler.

**Supervision:** Hakan Yolaçan.

**Validation:** Hakan Yolaçan.

**Visualization:** Hakan Yolaçan.

**Writing – original draft:** Hakan Yolaçan.

**Writing – review & editing:** Hakan Yolaçan, Serkan Güler.
